# Quantification of Significant Aortic Stenosis by Echocardiography versus Four-Dimensional Cardiac Computed Tomography: A Multi-Modality Imaging Study

**DOI:** 10.3390/diagnostics12123106

**Published:** 2022-12-09

**Authors:** Tom Kai Ming Wang, Ossama K. Abou Hassan, Zoran B. Popović, Brian P. Griffin, Luis Leonardo Rodriguez

**Affiliations:** Section of Cardiovascular Imaging, Heart, Vascular and Thoracic Institute, Cleveland Clinic, Cleveland, OH 44195, USA

**Keywords:** aortic stenosis, aortic valve, echocardiography, computed tomography, multimodality imaging

## Abstract

Transthoracic echocardiography (TTE) grading of aortic stenosis (AS) is challenging when parameters are discrepant, and four-dimensional cardiac computed tomography (4D-CCT) is increasingly utilized for transcatheter intervention workup. We compared TTE and 4D-CCT measures contributing to AS quantification. AS patients (*n* = 80, age 86 ± 10 years, 71% men) referred for transcatheter replacement in 2014–2017 were retrospectively studied, 20 each with high-gradient AS (HG-AS), classical and paradoxical low-flow low-gradient AS (CLFLG-AS and PLFLG-AS), and normal-flow low-gradient AS (NFLG-AS). Correlation and Bland–Altman analyses were performed between TTE and 4D-CCT parameters. There were moderate-to-high TTE versus 4D-CCT correlations for left ventricular volumes, function, mass, and outflow tract dimensions (r = 0.51–0.88), though values were mostly significantly higher by 4D-CCT (*p* < 0.001). Compared with 4D-CCT planimetry of aortic valve area (AVA), TTE estimates had modest correlation (r = 0.37–0.43) but were significantly lower (by 0.15–0.32 cm^2^). The 4D-CCT estimate of LVSVi lead to significant reclassification of AS subtype defined by TTE. In conclusion, 4D-CCT quantified values were higher than TTE for the left ventricle and AVA, and the AS subtype was reclassified based on LVSVi by 4D-CCT, warranting further research to establish its clinical implications and optimal thresholds in severe AS management.

## 1. Introduction

The main criteria used in guidelines to define severe aortic stenosis (AS) are primarily based on transthoracic echocardiography (TTE) Doppler measurements [1,2,3]. Patients with AVA < 1.0 cm^2^ or indexed AVA < 0.6 cm^2^/m^2^, mean gradient ≥ 40 mm Hg and peak velocity ≥ 4.0 m/s, all meeting severe AS criteria, have “high-gradient AS”. If there is discordance amongst these parameters, such as AVA meeting severe criteria (<1.0 cm^2^) but mean gradient and peak velocity not (<40 mm Hg and <4.0 m/s, respectively), then “low-gradient AS” is present. Low-gradient AS is usually sub-grouped into (1) classical low-flow low-gradient AS (CLFLG-AS) with a left ventricle stroke volume index (LVSVi) ≤ 35 mL/m^2^ and reduced left ventricular ejection fraction LVEF < 50%; (2) paradoxical low-flow, low-gradient AS (PLFLG-AS) with preserved LVEF ≥ 50%; and (3) normal-flow low-gradient (NFLG-AS) with LVSVi > 35 mL/m^2^ AS [2]. The need to accurately classify patients into these subgroups is because prognoses differ by subgroup; many patients with low-gradient AS have severe AS and would benefit from intervention, and discordant measurements may pose difficulties in how to manage such patients [4,5,6,7,8,9]. Confirming severe AS is critical, as aortic valve surgical or transcatheter interventions are recommended for severe aortic stenosis with at least one of the following, based on contemporary guidelines: symptoms, reduced LVEF, reduced exercise capacity or drop in blood pressure during exercise treadmill test, critical AS with peak velocity ≥ 5 m/s, brain natriuretic peptide biomarker > 3× upper limit of normal, rapid disease progression, or concomitant to other indicated cardiac surgery (in which case, moderate AS is sufficient for aortic valve intervention) [1,3].

Inaccurate measurements of left ventricular outflow tract (LVOT) diameters or velocities are the two main reasons for discordance in TTE. Methods suggested to address these include using a consistent LVOT diameter over time, measuring both LVOT diameter and LVOT velocity time integral (VTI) at the same position below the aortic valve, three-dimensional imaging of the LVOT, and multiple windows to obtain the highest possible aortic valve peak velocities and gradients [2]. As suggested by guidelines, there has been an increased usage of alternative imaging modalities to better quantify AS-related parameters, such as dobutamine stress echocardiography, transesophageal echocardiography, computed tomography and cardiac magnetic resonance techniques [10,11,12,13,14,15,16,17,18]. All of these modalities except for stress echocardiography include three-dimensional and multi-planar reconstruction techniques for more accurate anatomical measurements, such as for aortic valve planimetry. Aortic valve calcium scoring by the Agatston method on non-contrast gated computed tomography is also increasingly utilized for AS severity evaluation, with a score of ≥2000 in men and ≥1200 in women suggesting severe AS [2,19,20,21,22]. This is especially useful in low-gradient AS patients, where higher scores may highlight disease progression and adverse prognoses that are otherwise overlooked due to discrepant TTE-derived parameters [20,23]. While aortic valve calcium score calculations are also possible with contrast computed tomography, such techniques have not been standardized, and available literature is limited to a few observational studies [24,25]. Four-dimension contrast cardiac computed tomography (4D-CCT) using retrospective ECG-gating is an invaluable tool in the pre-procedural workup for transcatheter aortic valve replacement (TAVR), as it helps comprehensively characterize both cardiac and aortic morphometry for sizing and positioning of prosthetic aortic valves [1,3,26]. Despite 4D-CCT being routinely performed in pre-TAVR assessment, the agreements between 4D-CCT and TTE measures of AS severity and hemodynamics are not well established.

In this study, we aim to compare the measurements obtained from TTE and 4D-CCT for LVOT morphology, ventricular systolic function, and aortic valve properties. Furthermore, we demonstrate how these findings influence the AS subtype classification of patients planned to undergo TAVR.

## 2. Materials and Methods

### 2.1. Study Population

Patients with significant tri-leaflet AS, of calcific degenerative etiology by TTE, who underwent dedicated 4D-CCT within 6 months of TTE because of referral for TAVR during 2014–2017, were retrospectively identified. Eighty total cases were randomly selected, 20 each with high-gradient AS (HG-AS), CLFLG-AS, PLFLG-AS, and NFLG-AS subtypes based on TTE. Low or high flow was defined using the LVOT-stroke volume method by TTE (described below) indexed to body surface area (LVOT-SVi) ≤ or >35 mL/m^2^ respectively. Exclusion criteria encompassed aortic valve area > 1.2 cm^2^ by TTE (VTI continuity method), aortic valve morphology that was not trileaflet, history of valve surgery, at least moderate concurrent regurgitation or stenosis of another valve, and 4D-CCT performed beyond 6 months of TTE. Clinical characteristics including demographics, past medical history, and the Society of Thoracic Surgeons risk score were collected and calculated from the electronic medical record. The study was approved by our center’s Institutional Review Board with an informed consent waiver.

### 2.2. Echocardiography

Transthoracic echocardiography was performed using Vivid 7 or Vivid E9 (GE Healthcare, Milwaukee, WI, USA) or EPIQ 7C (Philips Medical Systems, Bothell, WA, USA) machines following standard institutional protocols and current American Society of Echocardiography guidelines for evaluation of aortic stenosis [2]. Left ventricular measurements included left ventricular end-diastolic volume (LVEDV), end-systolic volume (LVESV), stroke volume (using biplane method LVEDV − LVESV = LVSV, or using LVOT method of area multiplied by VTI, ie LVOT-SV), ejection fraction (LVEF), mass (LVM), LVOT diameter, LVOT area, and LVOT-VTI. Each measurement can be indexed to body surface area (such as LVOT-Svi). AVA by echo was determined using the three continuity equation methods: AVA = LVOT area × LVOT-VTI/aortic valve-VTI; AVA = LVOT area × LVOT peak velocity/aortic valvepeak velocity; or AVA = LVOT area × LVO—mean velocity/aortic valve mean velocity. All measurements were verified retrospectively by one of the study authors (OKAH) and re-measured as necessary.

### 2.3. Computed Tomography

**Figure 1:**
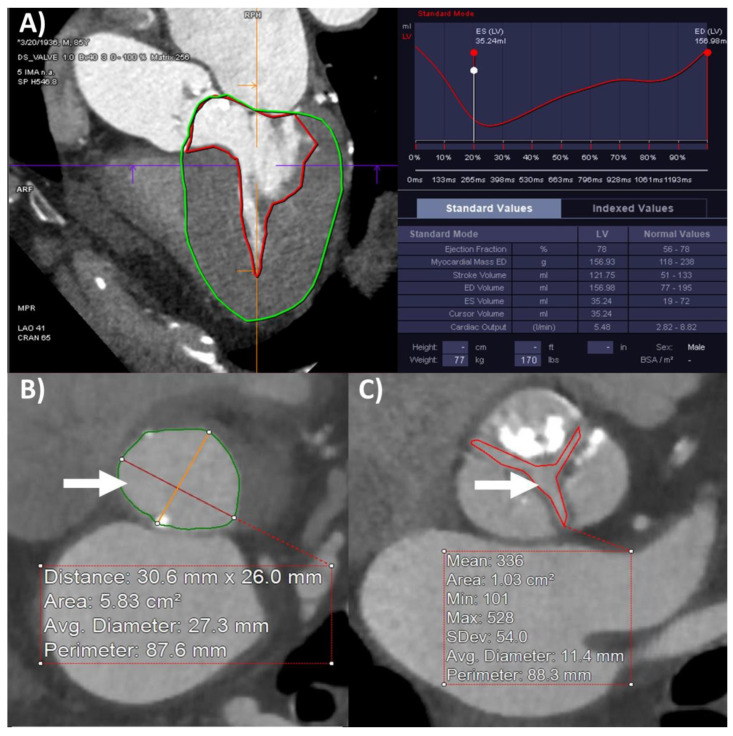
Cardiac computed tomography using four-dimensional retrospective ECG gating and iodinated contrast enhancement for aortic stenosis assessment. (**A**) Left ventricular dimensions end-diastolic (LVEDV), end-systolic (LVESV), and stroke (LVSV) volumes, ejection fraction (LVEF) and mass (LVM) measurements, (**B**) left ventricular outflow tract diameter and area (arrow, measured at 3.1 × 2.6 cm, 5.8 cm^2^), and (**C**) aortic valve area planimetry (arrow) measured at 1.0 cm^2^.

Computed tomography was performed using Siemens Somatom Definition Flash (2 × 128 slice) scanners (Siemens Healthcare, Erlangen, Germany). The TAVR-CT protocol was performed in all study patients. The 4D-CCT imaging was performed using retrospective ECG-gating and following iodinated contrast administration (with Omnipaque 300 or Omnipaque 350, by GE Healthcare, Milwaukee, WI, USA), to capture the whole heart with thin slices (0.75 mm) including the left ventricle and aortic valve for the entire cardiac cycle divided into 10 phases. This was followed by a non-gated high-pitch “Flash” or spiral acquisition of the chest, abdomen, and pelvis for thoracoabdominal aorta and pelvic arteries for vascular evaluation, though these images were not analyzed specifically for this study. The 4D-CCT analyses were performed using TeraRecon Aquarius iNtuition (Foster City, CA, USA) and Syngo Via Siemens Healthineers (Malvern, PA, USA) software at dedicated workstations retrospectively by study authors (T.K.M.W. and O.K.A.H.). LVEDV, LVESV, LVSV, LVEF, and LVM were measured on the 4D sequence with initial automated tracing of the left ventricle and subsequent manual corrections as necessary (Figure 1A). LVOT diameter and area were measured on the mid-systolic frame using multi-planar reconstruction (Figure 1B), while LVOT-VTI was calculated by LVSV divided by LVOT area. Anatomical planimetry of the in-plane AVA on 4D-CCT at peak systole using multi-planar reconstruction was used as the reference value (Figure 1C). All 4D-CCT measurements were performed retrospectively by a study author (T.K.M.W. or O.K.A.H.). 

### 2.4. Statistical Analyses

Data were presented as mean ± standard deviation for continuous variables and frequency (percentage) for categorical variables in the overall cohort and by AS subgroup. Univariable analyses between AS subgroups were compared using the post-hoc multiple-comparisons Bonferroni method for continuous variables and chi-squared tests for categorical variables, respectively. Correlations between TTE and 4D-CCT measurements of left ventricular and AVA parameters were assessed using scatterplots and Pearson correlation coefficients with *p*-values. Absolute and percentage differences or biases between TTE and 4D-CCT were assessed using Bland–Altman plots with 95% confidence intervals (95% CIs). Reclassification of LVSVi was assessed using a scatterplot of LVSVi bt 4D-CCT versus LVOT-SVi by TTE measurement. All tests were two-tailed and *p*-values < 0.05 were deemed statistically significant. Statistical analyses were performed using SPSS (version 24, IBM, Chicago, IL, USA) and Prism (version 8, GraphPad, San Diego, CA, USA) software. 

## 3. Results

Eighty AS patients undergoing both TTE and dedicated 4D-CCT were included, with clinical characteristics including AS subgroup displayed in Table 1. The mean age was 81 ± 9 years, 57 (71%) were male, and 76 (95%) were Caucasian; these characteristics were similar between AS subgroups. The commonest co-morbidities were hypertension in 68 (85%), coronary heart disease in 58 (73%, including 23 with myocardial infarction, 26 with prior coronary bypass grafting surgery and 25 with prior percutaneous coronary intervention), atrial fibrillation in 36 (45%), diabetes in 33 (41%), and peripheral artery disease in 28 (35%). Baseline clinical characteristics were similar across all groups, except for atrial fibrillation history being least common in the HG-AS subgroup. The overall mean STS score was 6.3 ± 4.6%, highest in the CLFLG-AS subgroup, and similar between the other three subgroups. 

Table 2 lists the TTE and 4D-CCT parameters of the cohort and AS subgroups. Based on their definitions, CLFLG-AS had lower LVEF than other subgroups, but it also had higher LVEDV and LVESV by both imaging modalities. CLFLG-AS and PLFLG-AS had lower LVOT-SVi (by TTE definition), lower LV-SV by 4D-CCT, and lower LVOT-VTI by TTE than the other two subgroups. Furthermore, associated with their definitions, NFLG-AS had higher AVA and dimensionless index on TTE than other subgroups, while HG-AS had higher aortic valve peak and mean velocities, gradients, and velocity-time integral than other subgroups. LVOT diameter and area, when measured by TTE, were similar across all groups, but were slightly higher for CLFLG-AS than the HG-AS and NFLG-AS subgroups when measured by 4D-CCT.

**Figure 2:**
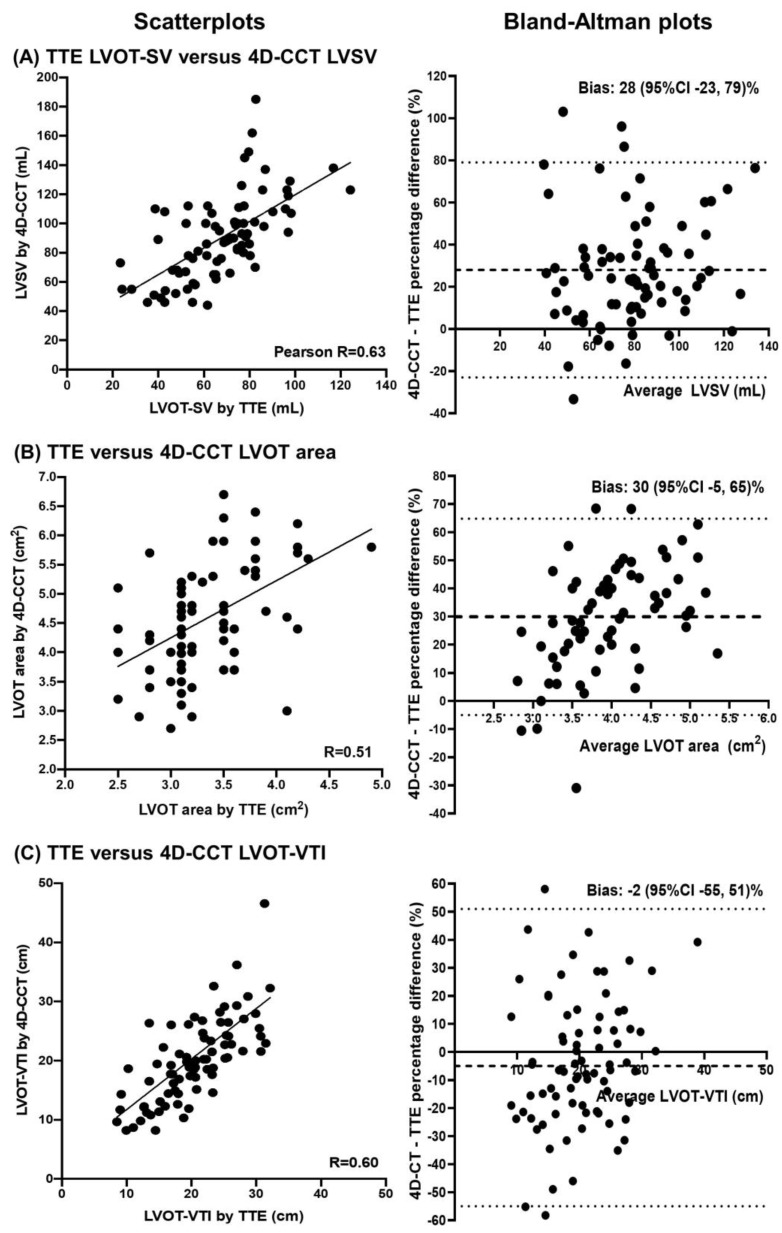
Comparisons between transthoracic echocardiography (TTE) and four-dimensional cardiac computed tomography (4D-CCT) estimates of left ventricular parameters important in aortic stenosis assessment. Scatterplots and Bland–Altman plots with Pearson correlation coefficients and percentage bias estimates with 95% confidence intervals (95% CI) comparing TTE and 4D-CCT measures of (**A**) left ventricular stroke volume (LVSV), (**B**) left ventricular outflow tract (LVOT) area, and (**C**) LVOT-velocity time integral (VTI).

Comparisons between TTE and 4D-CCT measurements of left ventricular parameters are presented in Table 3. The correlation was high for LVEDV, LVESV, and LVEF (r = 0.73–0.88), and moderate for LVM, LVOT diameter, area, and VTI (r = 0.51–0.60). LVSV by the TTE biplane method recorded a lower correlation (r = 0.33) than LVOT-SV by TTE method (r = 0.63) when compared to LVSV by 4D-CCT. However, all measures of LV volumes (by 21–60 mL), as well as LVOT diameter (by 0.4 cm) and LVOT area (1.2 cm^2^) were significantly larger by 4D-CCT than TTE. LVEF was also higher by 6% on 4D-CCT, while LVM was significant lower by 53 g on 4D-CCT. Notably, there were no significant differences in LVOT-VTI estimated by TTE and 4D-CCT (*p* = 0.771). Scatterplots and Bland–Altman plots of TTE versus 4D-CCT measures of LVSV, LVOT area, and LVOT-VTI are illustrated in Figure 2. The time differences between TTE and 4D-CCT scan dates had a median of 1 day (lower quartile 0, upper quartile 35 days), and there were no significant correlations between absolute differences in TTE versus 4D-CCT measurements of all parameters analyzed and the time differences between the two scans.

**Figure 3:**
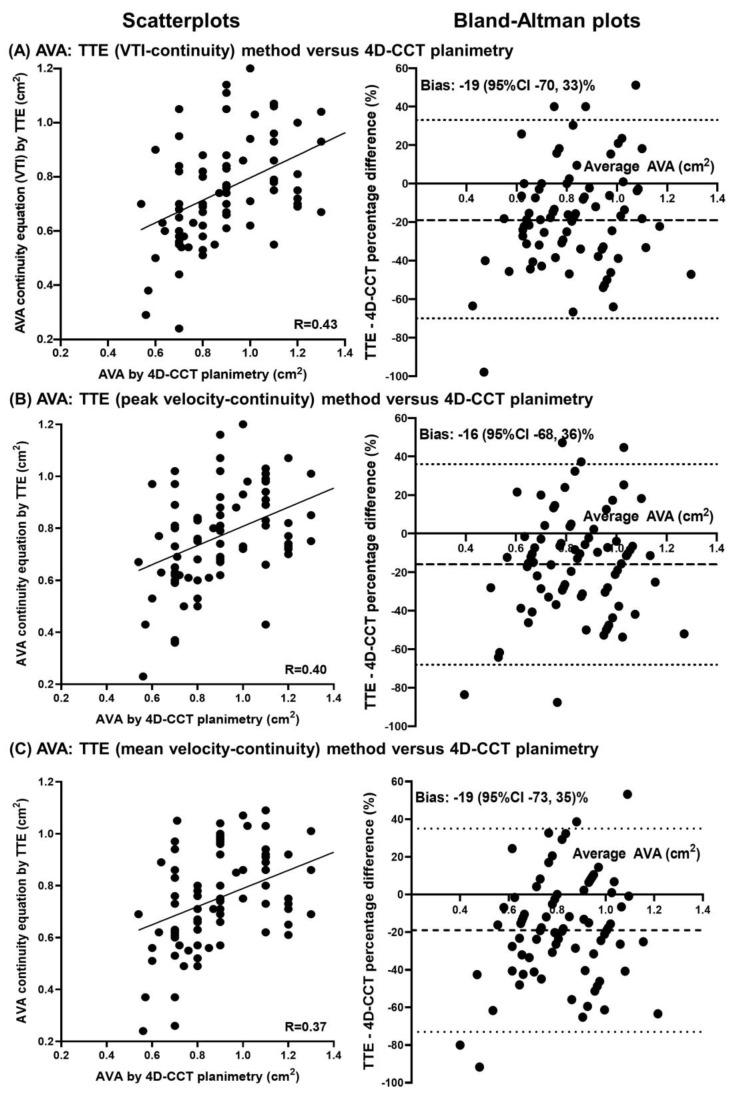
Comparisons between transthoracic echocardiography (TTE) and four-dimensional cardiac computed tomography (4D-CCT) estimates of aortic valve area (AVA). Scatterplots and Bland–Altman plots with Pearson correlation coefficients and percentage bias estimates with 95% confidence intervals (95% CI) comparing AVA measurements of 4D-CCT planimetry against TTE continuity equation methods using (**A**) velocity-time integrals (VTI), (**B**) peak velocities, and (**C**) mean velocities.

Table 4 lists the AVA measurements by TTE and 4D-CCT, using valve planimetry by 4D-CCT (cohort mean of 0.90 ± 0.21 cm^2^) as the reference standard. TTE estimates of the AVA using LVOT area and either VTI, peak, or mean LVOT and aortic valve velocities had modest correlations (r = 0.37–0.43), but were significantly smaller than AVA planimetry by 4D-CCT (by 0.13–0.15 cm^2^). As such, the percentages of patients with AVA ≤ 1.0 cm^2^ (threshold for severe by TTE) was 88–89% by TTE methods but only 71% using 4D-CCT planimetry. Scatterplots and Bland–Altman plots of TTE measures of AVA compared with 4D-CCT planimetry method are shown in Figure 3. 

**Figure 4:**
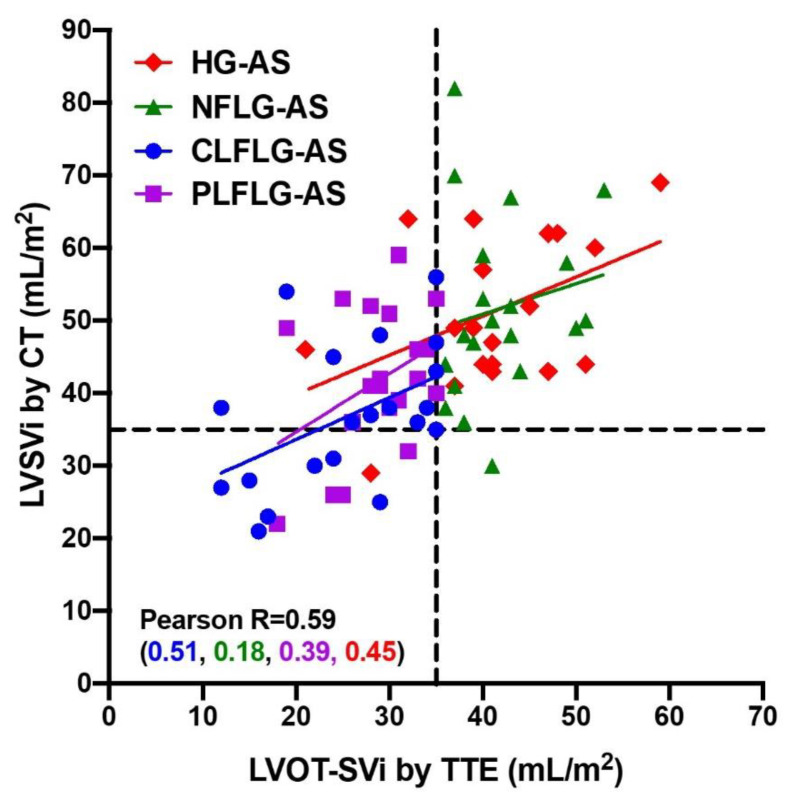
Correlations between transthoracic echocardiography (TTE) and 4-dimensional cardiac computed tomography (4D-CCT) of left ventricular stroke volume indexed for aortic stenosis classification. Scatterplot of left ventricular outflow tract stroke volume indexed (LVOT-SVi) by TTE against left ventricular stroke volume indexed (LVSVi) by 4D-CCT. Pearson correlation coefficient indicated for the whole cohort and subgroups of aortic stenosis (AS) cohort by TTE definitions: high gradient (HG-AS), classical and paradoxical low-flow low-gradient (CLFLG-AS and PLFLG-AS), and normal-flow low-gradient (NFLG-AS).

Figure 4 shows the scatterplot of LVOT-SVi by TTE compared with LVSVi by 4D-CCT, including by the four AS subgroups. There is a moderate correlation overall (r = 0.59), and in most of the subgroups (r = 0.39–0.51), but there is no correlation in the NFLG-AS subgroup (r = 0.18). In the 20 patients with C-LFLG and LVOT-SVi ≤ 35 mL/m^2^ by TTE, only 8 had LVSVi ≤ 35 mL/m^2^ by 4D-CCT, meaning 12 patients were re-classified as not having “low flow” if 4D-CCT was used for measuring LVSVi. In the 20 patients with P-LFLG and LVOT-SVi ≤ 35 mL/m^2^ by TTE, only 4 had LVSVi ≤ 35 mL/m^2^ by 4D-CCT, meaning 16 patients were re-classified as not having “low flow” if 4D-CCT was used for measuring LVSVi. 

## 4. Discussion

A significant minority of AS patients have discrepant TTE parameters, such as low gradient AS, and guidelines recommend multi-modality assessment in these scenarios, however there is paucity of literature comparing 4D-CCT and TTE in AS evaluation. This study uniquely compared the two imaging modalities with regards to left ventricular, outflow tract, and AVA measurements. 4D-CCT had significantly higher left ventricular parameters, especially volumes, than TTE, with moderate to high correlations, except for similar LVOT-VTI. For AVA, 4D-CCT planimetry was also significantly higher than TTE and therefore would affect severity grading if identical thresholds were used, with modest correlations. There were key differences in LVOT-SVi measurements by TTE and 4D-CCT that led to significant discrepancies in AS subtype classifications. 

For left ventricular analysis, 4D-CCT showed higher mean volumes and smaller mean mass than TTE, but otherwise moderate to high correlations between them. The amount of discrepancy is expected and similar to that between cardiac magnetic resonance and TTE chamber quantification [27]. For LVSV, when compared to 4D-CCT measurement, the TTE LVOT-SV method had a higher correlation and smaller discrepancy than the TTE LVEDV-LVESV method. This finding, together with contamination of mitral regurgitant volume when present and using the LVEDV-LVESV method, suggest that the use of the LVOT-SV method for AVA calculation is more likely to be accurate, and this is what is typically used in clinical practice. LVOT area was also significantly larger by 4D-CCT than TTE which is also well established. This, together with higher resolution of 4D-CCT, means many guidelines have moved towards recommending three-dimensional echocardiography, computed tomography, or magnetic resonance techniques to determine the LVOT and aortic annulus size, whether for AVA calculation or TAVR prosthesis sizing [1,10,26]. Uniquely, we found that LVOT-VTI estimated by 4D-CCT had not just moderate correlation but also a similar value to that calculated by TTE, therefore TTE derived LVOT-VTI can be used as a substitute in the calculations for LVSV and potentially AVA. 

As a centerpiece in AS severity grading, AVA can be determined using a variety of methods, such as planimetry of the anatomic orifice and the continuity equation for the effective orifice. Some prior studies have found, like ours, that 4D-CCT planimetry is larger than the TTE continuity equation method by 0.1–0.2 cm^2^. The anatomical area is expected to be larger than that measured at the vena contracta, and the difference between the anatomical and “physiological” orifice area are determined by factors such as the geometry of the individual valve orifice [28,29,30]. Based on these results, AVA measures are expected to be higher with 4D-CCT than TTE, suggesting that the AVA threshold for severe AS may need to be higher than the ≤ 1.0 cm^2^ used for TTE when 4D-CCT measures are utilized, otherwise the AS grade may be inappropriately classified as not being severe. Indeed, all the outcomes’ data on these various subgroups of AS in the literature are based on TTE rather than 4D-CCT measurements, which warrant further prognostic studies for 4D-CCT.

Significant differences exist in LVSVi estimates between TTE and 4D-CCT, even when the LVOT-SVi method was used for TTE. This has important implications when using LVSVi < 35 mL/m^2^ as the threshold for low flow [1,2]. As was shown, higher 4D-CCT values of LVSVi than TTE lead to a significant proportion of patients with low flow being reclassified as normal flow when the 35 mL/m^2^ threshold was utilized, regardless of what their AS severity, based on AVA, were. Classification of AS should be cautiously performed in this setting, and a separate and higher threshold for low flow developed for 4D-CCT is likely required. Other factors that provide complementary information to grade AS should be utilized when AS severity remains uncertain, including clinical presentation (especially AS-related symptoms) and other TTE parameters such as dimensionless index, aortic valve Agatston calcium score on non-contrast computed tomography, and dobutamine stress echocardiography findings [1,2,20].

This study has some limitations. It is a single-center retrospective observational study. In order to have the same number of patients with each of the four subtypes of AS, the overall sample size was 80 patients and therefore power was low. The intrinsic selection bias means only patients with significant AS who were referred for TAVR were included, so findings are not generalizable to less severe grades of AS, although narrowing the range of severity typically under-estimates the strength of correlation between modalities. A significant minority of patients had atrial fibrillation at time of TTE and/or 4D-CCT (*n* = 14, 18%), which may limit the accuracy of chamber and valve quantification by both imaging modalities, although excluding these patients did not significantly change the study findings. Patients with less-than-moderate mitral and aortic regurgitation were not excluded, and these lesions may affect biplane and sometimes LVOT stroke-volume calculations. Most patients did not have sufficient TTE image qualities to determine the AVA directly by planimetry for this to be analyzed. 4D-CCT does have inferior temporal resolution to TTE, and so accurate left ventricular ejection time by 4D-CCT could not be retrospectively derived. Furthermore, our TAVR 4D-CCT protocols are contrast-enhanced and therefore non-contrast aortic valve calcium scores were not analyzed. Other modalities for AS evaluation were not studied, such as transesophageal echocardiography and invasive catheterization for valve and stroke volume assessment. Finally, we did not assess patient outcomes to determine the prognostic value of 4D-CCT and optimal prognostic thresholds, which was not the focus of this cross-imaging correlation study and was also limited by study power.

## 5. Conclusions

In conclusion, our study sheds light on how 4D-CCT measures compare with TTE in the evaluation of AS, including in the AS subtypes with discrepant TTE findings. 4D-CCT and TTE have moderate-to-high correlations for the, left ventricle and modest correlations for AVA parameters. However, 4D-CCT had significantly higher values for most AS parameters, except LVOT-VTI, which was similar. Furthermore, the higher LVSVi estimated by 4D-CCT would reclassify a proportion of patients with low-flow AS that was classified by TTE. 4D-CCT therefore offers supplementary information to TTE in AS evaluation, especially when TTE is inconclusive, and further studies are necessary to evaluate the clinical implications of 4D-CCT derived AS parameters, and derive optimal AVA thresholds for severe AS specific to 4D-CCT.

## Figures and Tables

**Table 1:** Clinical characteristics of the cohort and aortic stenosis subgroup.

	Total	HG-AS	CLFLG-AS	PLFLG-AS	NFLG-AS	*p*-Value
Number of patients	80	20	20	20	20	
Demographics						
Age (years)	81 ± 9	79 ± 10	81 ± 9	82 ± 7	81 ± 12	
Male	57 (71%)	10 (50%)	16 980%)	17 (85%)	14 (70%)	0.071
Caucasian	76 (95%)	17 (85%)	19 (95%)	20 (100%)	20 (100%)	0.421
Past medical history						
Hypertension	68 (85%)	19 (95%)	17 (85%)	17 (85%)	15 (75%)	0.371
Diabetes	33 (41%)	8 (40%)	9 (45%)	10 (50%)	6 (30%)	0.614
Atrial fibrillation	36 (45%)	4 (20%)	10 (50%)	14 (70%)	8 (40%)	**0.015**
Coronary artery disease	58 (73%)	10 (50%)	18 (90%)	14 (70%)	16 (80%)	0.061
Myocardial infarction	23 (29%)	5 (25%)	4 (20%)	8 (40%)	6 (30%)	0.545
Coronary artery bypass grafting	26 (33%)	5 (25%)	7 (35%)	7 (35%)	7 (35%)	0.877
Percutaneous coronary intervention	25 (31%)	3 (15%)	8 (40%)	7 (35%)	7 (35%)	0.330
Cardiac implantable electronic device	15 (19%)	1 (5%)	6 (30%)	4 (20%)	4 (20%)	0.242
Stroke	14 (18%)	5 (25%)	2 (10%)	5 (25%)	2 (10%)	0.374
Peripheral arterial disease	28 (35%)	6 (30%)	8 (40%)	5 (25%)	9 (45%)	0.532
Chronic kidney disease	17 (21%)	3 (15%)	5 (25%)	2 (10%)	7 (35%)	0.221
Dialysis	2 (3%)	0 (0%)	1 (5%)	0 (0%)	1 (5%)	0.562
Chronic lung disease	10 (13%)	2 (10%)	2 (10%)	4 (20%)	2 (10%)	0.712
Cirrhosis	2 (3%)	0 (0%)	0 (0%)	0 (0%)	2 (10%)	0.104
STS score (%)	6.3 ± 4.6%	4.8 ± 1.9% *	9.1 ± 6.9% *	5.7 ± 3.7%	5.5 ± 3.4%	

High gradient (HG-AS), classical and paradoxical low-flow low-gradient (CLFLG-AS and PLFLG-AS), and normal-flow low-gradient (NFLG-AS) aortic stenosis. Numbers are mean ± standard deviation or frequency (percentage). Chi-square *p*-values for categorical variables are shown in the last column, and bold means *p*-value < 0.05. Pairs labelled as * in the same row for continuous variables indicate significant difference with *p*-value < 0.05 on Bonferroni method post-hoc multiple comparisons tests.

**Table 2:** Echocardiography and computed tomography parameters of the cohort and aortic stenosis subgroup.

	Total	HG-AS	CLFLG-AS	PLFLG-AS	NFLG-AS
	80	20	20	20	20
Transthoracic echocardiography					
Left ventricular end-diastolic volume (mL)	101 ± 39	96 ± 30 *	135 ± 43 *^’	84 ± 19 ^	88 ± 35 ’
Left ventricular end-systolic volume (mL)	49 ± 32	38 ± 13 *	88 ± 38 *^’	36 ± 12 ^	37 ± 18 ’
Left ventricular stroke volume (mL)	51 ± 17	59 ± 20	48 ± 15	48 ± 11	51 ± 20
Left ventricular outflow tract stroke volume (mL)	68 ± 20	79 ± 16 *’	50 ± 15 *^	60 ± 12 ’^#^	81 ± 14 ^^#^
Let ventricular ejection fraction (%)	54 ± 13%	61 ± 6% *	37 ± 10% *^’	58 ± 8% ^	59 ± 9% ’
Left ventricular mass (g)	207 ± 64	193 ± 62	240 ± 68	203 ± 58	191 ± 60
Left ventricular outflow tract diameter (cm)	2.1 ± 0.1	2.1 ± 0.2	2.1 ± 0.1	2.1 ± 0.1	2.1 ± 0.1
Left ventricular outflow tract area (cm^2^)	3.4 ± 0.5	3.3 ± 0.6	3.4 ± 0.5	3.4 ± 0.4	3.3 ± 0.5
Left ventricular velocity-time integral (cm)	20 ± 6	24 ± 5 *’	14 ± 4 *^	18 ± 4 ’^#^	25 ± 4 ^^#^
Aortic valve velocity-time integral (cm)	91 ± 22	114 ± 24 *^’	81 ± 13 *	80 ± 11 ^	89 ± 16 ’
Aortic valve mean velocity (cm/s)	279 ± 47	341 ± 48 *^’	254 ± 27 *	257 ± 22 ^	265 ± 14 ’
Aortic valve peak velocity (cm/s)	388 ± 55	462 ± 55 *^’	358 ± 29 *	360 ± 26 ^	371 ± 19 ’
Aortic valve mean gradient (mmHg)	32 ± 12	47 ± 14 *^’	26 ± 5 *	27 ± 5 ^	28 ± 3 ’
Aortic valve peak gradient (mmHg)	62 ± 19	87 ± 21 *^’	51 ± 9 *	52 ± 8 ^	56 ± 8 ’
Dimensionless index	0.23 ± 0.06	0.22 ± 0.06 *	0.18 ± 0.04 ^’	0.23 ± 0.05 ^^#^	0.29 ± 0.06 *’^#^
Aortic valve area (velocity time integral continuity method cm^2^)	0.76 ± 0.20	0.72 ± 0.17 *	0.61 ± 0.14 ^’	0.76 ± 0.16 ^^#^	0.93 ± 0.20 *’^#^
Computed tomography					
Left ventricular end-diastolic volume (mL)	160 ± 55	147 ± 36 *	216 ± 69 *^’	132 ± 27 ^	147 ± 37 ’
Left ventricular end-systolic volume (mL)	70 ± 55	46 ± 17 *	145 ± 63 *^’	45 ± 13 ^	45 ± 12 ’
Left ventricular stroke volume (mL)	90 ± 28	100 ± 26 *	71 ± 20 *^	87 ± 24	102 ± 32 ^
Let ventricular ejection fraction (%)	59 ± 17%	69 ± 9%*	35 ± 10% *^’	65 ± 9% ^	69 ± 9% ’
Left ventricular mass (g)	154 ± 34	160 ± 33	170 ± 38	142 ± 29	145 ± 32
Left ventricular outflow tract diameter (cm)	2.5 ± 0.2	2.4 ± 0.3 *	2.6 ± 0.2 *^	2.5 ± 0.2	2.4 ± 0.2 ^
Left ventricular outflow tract area (cm^2^)	4.6 ± 0.9	4.4 ± 1.1 *	5.2 ± 0.7 *^	4.8 ± 0.7	4.0 ± 0.8 ^
Left ventricular velocity-time integral (cm)	21 ± 8	23 ± 5 *	14 ± 4 *^	19 ± 5 ’	26 ± 11 ^’
Aortic valve area (planimetry cm^2^)	0.90 ± 0.21	0.86 ± 0.24	0.80 ± 0.20 *	0.96 ± 0.18	0.99 ± 0.18 *

High-gradient (HG-AS), classical and paradoxical low-flow low-gradient (CLFLG-AS and PLFLG-AS), and normal-flow low-gradient (NFLG-AS) aortic stenosis. Numbers are mean ± standard deviation or frequency (percentage). Pairs labelled as *,^, ’ and ^#^ in the same row indicate significant *p*-value < 0.05 on Bonferroni method post-hoc multiple comparisons tests.

**Table 3:** Comparisons and correlations between echocardiography and computed tomography measures of left ventricular and outflow tract parameters.

Parameter	Echocardiography	Computed Tomography	Difference (Paired *t*-Test) (95% CI, *p*-Value)	Correlation r (*p*-Value)
Left ventricular end-diastolic volume (mL)	101 ± 39	160 ± 55	**60 (51, 68; <0.001)**	**0.734 (<0.001)**
Left ventricular end-systolic volume (mL)	49 ± 32	70 ± 55	**21 (14, 28; <0.001)**	**0.878 (<0.00)**
Left ventricular stroke volume (mL)	51 ± 17	90 ± 28	**39 (33, 45; <0.001)**	**0.327 (0.003)**
Left ventricular outflow tract stroke volume (mL)	68 ± 20		**23 (18, 27; <0.001)**	**0.631 (<0.001)**
Let ventricular ejection fraction (%)	54 ± 13%	59 ± 17%	**6 (3, 8; <0.001)**	**0.759 (<0.001)**
Left ventricular mass (g)	207 ± 64	154 ± 34	**−53 (−65, −41; <0.001)**	**0.513 (<0.001)**
Left ventricular outflow tract diameter (cm)	2.1 ± 0.1	2.5 ± 0.2	**0.43 (0.38, 0.47; <0.001)**	**0.518 (<0.001)**
Left ventricular outflow tract area (cm^2^)	3.4 ± 0.5	4.6 ± 0.9	**1.2 (1.1, 1.4; <0.001)**	**0.507 (<0.001)**
Left ventricular outflow tract velocity-time integral (cm)	20 ± 6	21 ± 8	0.2 (−1.3, 1.7; 0.771)	**0.603 (<0.001)**

95% CI = 95% confidence interval. Bold means *p*-value < 0.05.

**Table 4:** Comparisons and correlations between echocardiography and computed tomography estimates of aortic valve area.

AVA (cm^2^)	Measurement	% of Patients AVA ≤ 1.0 cm^2^	Difference (95% CI, *p*-Value) Versus CT Planimetry	Correlation (*p*-Value) Versus CT Planimetry
CT: valve planimetry	0.90 ± 0.21	57 (71%)		
TTE: LVOT area, LVOT-VTI, AV-VTI	0.76 ± 0.20	70 (88%)	−0.15 (−0.10, −0.19; <0.001)	0.432 (<0.001)
TTE: LVOT area, LVOT-Vmax, AV-Vmax	0.77 ± 0.19	70 (88%)	−0.13 (−0.08, −0.18; <0.001)	0.401 (<0.001)
TTE: LVOT area, LVOT-Vmean, AV-Vmean	0.75 ± 0.20	71 (89%)	−0.15 (−0.10, −0.20; <0.001)	0.369 (0.001)

AVA = aortic valve area, 95% CI = 95% confidence interval, CT = computed tomography, TTE = transthoracic echocardiography, LVOT = left ventricular outflow tract, VTI = velocity time integral, AV = aortic valve, Vmax = peak velocity, Vmean = mean velocity.

## Data Availability

The data presented in this study are available in the article.
